# Applying Implementation Science to Secure a Sustainable Supply of UNIMMAP MMS for National Antenatal Care Services in Indonesia

**DOI:** 10.3390/nu18132162

**Published:** 2026-07-03

**Authors:** Holis Abdul Holik, Otte Santika, Auliya Suwantika, John Atwater, Jarno de Lange, Abdul Razak Thaha, Endang Laksminingsih Achadi, Hera Nurlita, Erni Rahmawati, Rimbawan, Clayton Ajello

**Affiliations:** 1 Center of Excellence for Pharmaceutical Care Innovation (PHARCI), Faculty of Pharmacy, Padjadjaran University, Jl. Ir Soekarno KM 21, Jatinangor Sub-District, Sumedang Regency 45363, West Java Province, Indonesia; holis@unpad.ac.id (H.A.H.);; 2 The Vitamin Angel Alliance, Indonesia, Ruko Sentul Tower Apartment (STA) Niaga Blok B No. 2, Citaringgul, Babakan Madang, Bogor Regency 16810, West Java Province, Indonesia; osantika@vitaminangels.org; 3 Ataqua Regulatory Services, 20878 Blue Ridge Mountain Road, Paris, VA 20130, USA; jba@ataquars.com; 4 The Vitamin Angel Alliance, 6500 Hollister Ave Suite 130, Goleta, CA 93117, USA; jdelange@vitaminangels.org; 5 Indonesian MMS-TAG, Indonesian Nutrition Initiative (IGI), Kompleks Bappenas A1 Jl. Siaga Raya Pejaten, Jakarta Selatan City 12510, Jakarta Province, Indonesia; 6 Indonesian Center for Nutrition Studies, Faculty of Public Health, Hasanuddin University, Jl. Perintis Kemerdekaan No. KM.10, Tamalanrea Indah, Kec. Tamalanrea, Makassar City 90245, South Sulawesi Province, Indonesia; 7 Center for Family Welfare Research (PUSKA), Faculty of Public Health, Universitas Indonesia, Building G No. 210, Depok City 16424, West Java Province, Indonesia; 8 Directorate of Family Health Service, Ministry of Health, H.R. Rasuna Said Road Blok X-5 Kavling 4-9, Jakarta Selatan City 12950, Jakarta Province, Indonesia; 9 Directorate of Traditional Medicine, Health Supplement and Cosmetic Standardization, The Indonesian Food and Drug Authority of The Republic of Indonesia (BPOM), Bhinneka Tunggal Ika Building, 2nd Floor, Jl. Percetakan Negara No. 23, Jakarta 10560, Jakarta Province, Indonesia; erni.rahmawati@pom.go.id; 10 Department of Community Nutrition, Faculty of Human Ecology, IPB University, FEMA Building W1–L2, Jl. Kamper, IPB Dramaga Campus, Bogor Regency 16680, West Java Province, Indonesia; 11 Kirk Humanitarian, 2755 E. Cottonwood Parkway, Suite 450, Salt Lake City, UT 84121, USA

**Keywords:** implementation science, implementation research, MMS, UNIMMAP, manufacturing, securing a sustainable supply of MMS, expert consensus specification

## Abstract

Background: The United Nations International Multiple Micronutrient Antenatal Preparation of a multiple micronutrient supplement (UNIMMAP MMS or MMS) is replacing iron and folic acid supplementation (IFAS) in antenatal care (ANC) in low- and middle-income countries (LMICs). An investigation into determining how to secure a sustainable supply of MMS began in response to the Indonesian Ministry of Health (MOH)’s decision to introduce MMS into its national health services. Objective: We aimed to identify and test sustainable strategies for securing MMS supplies. Methods: A three-phase implementation science framework was applied to (1) foster an enabling environment for securing MMS supplies, (2) undertake implementation research (IR) to compare sourcing strategy options, and (3) plan and execute actions to scale MMS supply availability and distribution. The MOH assumed ownership of the initiative and guided policy, procurement, and program decisions. Results: (1) Landscaping resulted in recommendations that triggered supply-related policies, an accommodating regulatory framework, integration of MMS into key government support systems (i.e., budget, finance, procurement, and distribution), and identification of supply strategy options. (2) IR resulted in the selection of a local manufacturing and sourcing strategy for acquiring a sustainable supply of high-quality MMS product while retaining an option to import a limited supply of MMS during scaling. (3) A multi-year plan was developed to scale MMSs within ANC services. Conclusions: Applying implementation science provided an evidence-based framework with which to identify, establish, and test a sustainable strategy for securing MMS supplies and yielded insights useful for other countries introducing MMS into their national health systems.

## 1. Introduction

Multiple-micronutrient deficiencies during pregnancy are a public health problem for women in low- and middle-income countries (LMICs). They lead to poor maternal health and adverse pregnancy outcomes, including low birth weight, pre-term birth, still birth, and poor child growth and development. Antenatal supplementation with iron and folic acid (IFAS) has been the standard of antenatal care (ANC) for decades. However, there is an ongoing shift in momentum in antenatal supplementation from IFAS to the United Nations International Multiple Micronutrient Antenatal Preparation (UNIMMAP) Multiple Micronutrient Supplement (hereafter, MMS means UNIMMAP MMS) [1]. MMS was developed by the World Health Organization (WHO), UNICEF, and the United Nations University in 1999 [2] to provide iron, folic acid, and 13 other vitamins and minerals in one formulation. MMS is more effective than IFAS in addressing micronutrient deficiency and its adverse consequences for maternal health and pregnancy outcomes [3,4,5]. The WHO updated its guidelines for a positive pregnancy experience in 2020, revising MMS use from “not recommended” to “recommended in the context of rigorous research”, including IR [6], and added MMS to the WHO Model List of Essential Medicines in 2021 [7].

In 2020, the government and other key stakeholders in Indonesia reached a consensus, indicating that (1) micronutrient deficiency is a public health problem in Indonesia [8,9,10]; (2) MMS is effective, safe, and cost-effective; (3) implementation strategies that ensure effective delivery of MMS and a secure supply of it should be designed and tested to support the transition from IFAS to MMS use. To translate this consensus into action, the Indonesian MMS Technical Advisory Group (MMS-TAG) was established, hosted by the Indonesian Nutrition Initiative (IGI), and recognized by the MOH to help bridge research and policy, explore MMS use in Indonesia, and provide recommendations for its integration into ANC services [11] (Figure S1).

The MMS-TAG established two key questions: (1) Could low rates of adherence among pregnant women using IFAS be avoided with use of MMS (described elsewhere [11]), and (2) Could the MOH secure a sustainable supply of MMS, and, if so, how could this be achieved? This paper describes how implementation science (IS) was applied to secure a sustainable *supply* of MMS.

## 2. Materials and Methods

### 2.1. Implementation Science (IS) Framework

**Figure 1:**
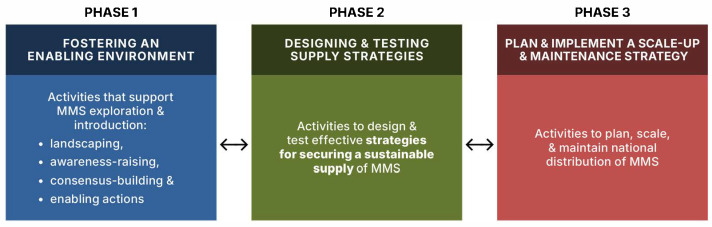
Implementation Science Framework.

An IS approach was used to identify, test, and implement a strategy for securing MMS supplies for ANC services (Figure 1). Each IS activity performed to secure MMS supplies between 2019 and 2024 was recorded and later ranked and presented as an enablement track to reflect the relative strength of its contribution to the acquisition of a sustainable supply (See Figure S1). Methods for enablement tracking and assigning ratings were adapted from those used during the introduction of MMS in another study [12].

### 2.2. Phase 1: Fostering an Enabling Environment

#### 2.2.1. Overall Landscape Analysis

A Supply Context Assessment (SCA) tool, adapted and expanded from the Supply Readiness Assessment (SRA) [13] tool, was developed. It includes four modules that examine (1) national supply-related policies, (2) MMS product regulatory requirements, (3) support systems (i.e., budget, finance, procurement, and distribution), and (4) manufacturers/suppliers. These modules were implemented between January 2020 and July 2022.

The SCA modules derived information from government publications and unpublished reports, individual interviews, and group discussions. Individual interviews (*n* = 8) were conducted with key informants from the MOH. Separate group discussions were conducted with stakeholders from (1) the industry and the Indonesian Food and Drug Authority (BPOM) (*n* = 8); (2) universities, regulatory and policy experts from the government, and manufacturing experts from the industry (*n* = 14); (3) PHARCI, BPOM, MOH, technical organizations, and manufacturers (*n* = 36).

Semi-structured interview guides with pre-defined questions and example probes were used in the individual interviews. Focus group discussions (FGDs) were held to collect information and generate consensus recommendations on supply-related enabling activities. The FGD guides included topics for inquiry, while allowing for clarification and discussion; but also provided for an opportunity for open-ended discussion.

#### 2.2.2. Comprehensive Economic Evaluations

Additional methods were adapted and used to confirm (1) the cost-effectiveness and costs vs. benefits of MMS adoption using the Micronutrient Initiative’s tool, which was customized for the Indonesian context [14,15], and (2) budget impact. These two analyses were used to determine the affordability of MMS adoption considering various sourcing and transition scenarios from IFAS to MMS.

#### 2.2.3. Specialized Assessment of Manufacturers

An initial, brief pre-qualification questionnaire and a subsequent detailed questionnaire were designed by manufacturing/procurement experts. They were modeled after similar questionnaires used by purchasers of pharmaceutical products, including UNICEF [16], and implemented to determine whether local manufacturers were likely to be capable of and interested in manufacturing UNIMMAP MMS.

*Initial pre-qualification.* The initial pre-qualification questionnaire used objective indicators against which manufacturers’ compliance was assessed (0 = yes, and 1 = no), as described in Table 1. With the assistance of the national manufacturers’ associations of Indonesia, the universe of potential pharmaceutical manufacturers in Indonesia was identified and surveyed (*n* = 38), and a subset were interviewed via videoconference (*n* = 8).

*In-depth manufacturer pre-qualification*. The detailed pre-qualification questionnaire was sent to initially pre-qualified, short-listed manufacturers (*n* = 8) to determine (1) what good manufacturing practices (GMPs) they followed, (2) what GMP audit certificates they held, (3) their annual tablet-manufacturing capacity, and (4) their ability to manufacture MMS according to internationally recognized pharmacopeial standards and GMP requirements for pharmaceutical products described in the internationally recognized *Expert Consensus on an Open-Access UNIMMAP MMS Product Specification, version 2020* [17] and the updated *version 2024* (Consensus Specification) [18,19].

#### 2.2.4. Generating Consensus Based on SCA Results

The MMS-TAG transformed information generated from the SCA into consensus recommendations for the MOH through a multi-step process. Information generated was first reviewed, discussed, and deliberated upon by the MMS-TAG to develop preliminary conclusions/recommendations and policy briefs. Subsequently, further MMS-TAG meetings were convened, with invitations sent to stakeholders from the MOH (including the Minister of Health), PHARCI, subject-matter experts/academics, and BPOM to discuss evidence, consider policy issues and potential solutions, or derive further input from the MOH. MMS-TAG recommendations were then finalized by the 10-member MMS-TAG through consensus and presented to the Minister of Health for follow-up action.

To be clear, the international donor for this investigation was not a party to operational decision-making performed by the MMS-TAG or the MOH and did not participate in discussions or deliberations pertaining to any recommendations. The lead implementing partner acted as an administrator, contractor, and coordinator of activities on behalf of the MMS-TAG and/or the MOH but was not part of MMS-TAG deliberations pertaining to recommendations. When acting as technical advisors in program activities, all technical partners generated information and delivered advice to the MMS-TAG, but none participated in making recommendations, a process carried out by the MMS-TAG. This separation of information gathering and recommendation-making minimized the effects of bias on the recommendations and actions.

### 2.3. Phase 2: Designing and Testing Supply Strategies

To examine, compare, and test the feasibility of the options for local manufacturing versus importing MMS, two activities were carried out by international technical implementing partners with the support of Indonesian stakeholders and financing from a U.S.-based foundation.

*Examining Indonesian-based manufacturers’ capacity for MMS production.* Based on the results obtained from manufacturer assessments, qualified manufacturers were invited to respond to an RFP. The RFP required submission of (1) specific MMS product representations and warranties (8 items), (2) a timeline for delivery (0 = no; 1 = yes), and (3) a valid proposal to produce MMS conformant with the Consensus Specification at a specified price and volume (0 = no; 1 = yes). Manufacturers unable to provide all specific product representations, a realistic timeline for delivery (within 36 months), and a proposal that fully met the technical requirements of the Consensus Specification were disqualified. The remaining manufacturers’ price proposals were then reviewed. The RFP instructions informed all manufacturers that only one contract would be awarded after contract negotiations.

*Examining requirements for MMS importation.* One qualified manufacturer already selling MMS in the global marketplace was engaged to examine the feasibility of selling MMS to the MOH in (1) finished consumer packaging and (2) bulk for local bottling and labeling in consumer packaging. Either option was viable for the MOH’s procurement agency. The selected manufacturer prepared a plan identifying the steps of selling MMS to the MOH and importing it. This option was explored to serve as a backup plan in case local manufacturing could not be achieved and/or took longer than expected to scale up.

### 2.4. Phase 3: MMS-Supply-Scaling Plan and Implementation

During phase 3, with guidance from the MOH, technical implementing partners developed a consensus-driven, multi-year, phased, and costed scale-up plan to secure MMS supplies for recommendation to the MOH. The plan was informed by the results of IS phases 1 and 2 and consistent with national, geographic scaling. The MOH delegated the assembly of a final plan to PHARCI.

## 3. Results

### 3.1. Phase 1: Fostering an Enabling Environment

Table 2 presents results derived from information generated during implementation of the SCA for three of four supply contexts (i.e., policy, regulatory, and manufacturer/supplier), including themes identified and associated within each context, findings associated with each thematic area, MMS-TAG consensus recommendations communicated to the MOH, and MOH enabling actions/outputs achieved.

#### 3.1.1. Supply Policy Context

The results obtained from landscaping the supply policy context (Table 2, Section 2.1) revealed four themes that needed to be overcome to introduce and scale MMS use in Indonesia: (1) the lack of policies permitting MMS procurement/access, (2) the absence of MMS in the Indonesian Essential Medicines List (IEML) and the Indonesian Formulary (IF), (3) the lack of a formal designated classification of MMS, and (4) the absence of appropriate policies for MMS use in ANC services.

#### 3.1.2. Supply Regulatory Context

The results obtained from landscaping the supply regulatory context (Table 2, Section 2.2) revealed two themes that needed to be overcome to introduce and scale MMS use in Indonesia: (1) The internationally recognized formulation of MMS is at variance with the Indonesian Pharmacopeia regarding the daily intake allowances for selenium, with the Indonesian standard being 5mcg less than the UNIMMAP formulation standard at 65mcg. (2) International MMS quality and GMP standards are at variance with Indonesia’s regulatory standards for health supplements regarding which index vitamins must be tested and BPOM’s lack of a requirement to conduct dissolution testing [24].

#### 3.1.3. Support Systems (Budget, Finance, Procurement, and Distribution) Context

*Budget and finance systems.* The results of landscaping the budget and finance systems revealed that determining how to incorporate the cost of a new intervention into the MOH’s budget would be challenging because these systems’ processes are often opaque and involve multiple decision-makers across various ministries. The key theme identified was the absence of a bridge between different approaches used by the MOH and Ministry of Finance (MOF) for decision-making. The MOH primarily depends upon cost–benefit analysis to select interventions with which to improve health services. The MOF focuses on the incremental costs of an intervention and its implementation. Both agencies found that affordability provided common ground. MOH decision-makers focused on preparing a cost–benefit computation and a budget impact assessment to reflect how affordability serves as the bridge to budget allocation in negotiations with the MOF, as summarized below:

- Cost effectiveness and cost–benefit analyses [14,15]: The cost of consumer-packaged MMS procured in Indonesia was projected to be in the range of USD 2.50 per pregnancy (i.e., a 180-count bottle) depending upon the volume purchased and the level of prepayment provided, which, in Indonesia, is on par with the cost of IFAS. The cost–benefit ratio of MMS for Indonesia was found to be 483 (reflecting a very favorable cost–benefit ratio, even though the relatively high price quoted in the UNICEF supply catalog in 2020, namely, USD 3.27 per pregnancy, was used) [25]. This places MMS among the most cost-effective interventions within Indonesian maternal and child health services.
- Budget impact analysis [26]: It was estimated that adding MMS to ANC services would not increase the existing MOH budget. The budget impact analysis included various scenarios for introducing MMS into ANC services, suggesting that the MOH (1) could affordably scale MMS over a three-to-five-year period as well as (2) begin procurement of MMS and assume full responsibility for financing all procurement over a three- or five-year period, but (3) it would need to depend on some level of donated MMS supplies in each of the first 3 years of the scale-up period while local manufacturing was scaled to meet national needs, thus eventually eliminating the need for donated product.
- Affordability: Given the high priority the Government of Indonesia has placed on improving maternal health status and pregnancy outcomes, implementing MMS was deemed affordable whether based on the price of MMS and transition costs or on a cost-effectiveness ratio [27].

*Procurement and distribution systems*: Full landscaping of procurement and distribution systems was deemed unnecessary because sub-national governmental jurisdictions across Indonesia utilize a national e-catalog ordering and delivery system that is generally recognized to operate effectively and efficiently. The MOH-operated e-catalog system automatically incorporates medicinal products once the MOH issues a tender for the product. Further efforts are needed/planned to gain incremental efficiencies.

#### 3.1.4. Manufacturer/Supplier Context

The results obtained from landscaping the manufacturing/supplier context revealed two overarching themes: enablers of and barriers to manufacturing (Table 2, Section 2.3) that needed to be overcome or exploited to introduce and scale MMS use in Indonesia. These enablers and barriers suggested that local manufacturing was feasible but would be years away from achieving requisite manufacturing capacity to meet national needs, and scaling up MMS use would require an extended transition period during which the MOH would need to procure imported MMS or depend on imported donated MMS. Landscaping yielded insights into sourcing and the universe of manufacturers.

*MMS-sourcing options:* The investigators identified local MMS manufacturing and importation as two viable options for securing MMS supplies. Legal requirements favored manufacturing in Indonesia. However, specific obstacles that precluded any direct procurement of MMS by the MOH to test local capability were identified:

- Market obstacles: Local manufacturers resist beginning costly product development for a low-margin, public sector product in the absence of a procurement agreement.
- Regulatory obstacles: Local manufacturers are unwilling to engage in contract negotiations when the codified regulatory requirements required to help one understand how to produce a product that could be market-authorized for use in Indonesia are not in place.
- Governmental authority obstacles: Indonesian governmental entities cannot enter into a procurement agreement with any manufacturer without an approved regulatory framework for the product.

Concurrent with this investigation, UNICEF and two foundations were collaborating to catalyze a global network of regional manufacturers, and Indonesia was already targeted by these organizations as a site for exploring MMS manufacturing. To overcome obstacles impeding direct procurement of MMS by the MOH, foundation funding was secured to contract a manufacturer to test local ability to produce MMS for export in accordance with the Consensus Specification.

*Universe of foreign and local manufacturers:* The SCA module on manufacturer/supplier context determined that manufacturers outside of Indonesia (*n* = 3) sold MMS in the global marketplace in 2020 but had limited production capacity and that Indonesian manufacturers (*n* = 38) may be able to manufacture MMS. Many Indonesian manufacturers (*n* = 22) were eliminated from further consideration after initial pre-qualification because they either did not have product lines compatible with production of a health supplement manufactured as a pharmaceutical product or did not respond to multiple inquiries about their capabilities and qualifications. Others were eliminated (*n* = 2) because they focused only on private-sector sales. Among the remaining manufacturers (*n* = 14), a limited number were deemed generally capable of and interested in manufacturing MMS (*n* = 8).

### 3.2. Phase 2: Designing and Testing Strategies for Securing a Sustainable MMS Supply

#### 3.2.1. Sourcing MMS from Local Manufacturers

On the basis of the results obtained from the SCA manufacturer/supplier context module, an RFP process was designed and implemented and then opened to a subset of fully qualified manufacturers (*n* = 8). This process lasted approximately 8 weeks, including opportunities for manufacturers to ask questions and clarify RFP requirements. Technical and price proposals were assessed separately. Four of eight submissions were deemed responsive. Two of four manufacturers were selected based on full compliance with the technical requirements and good performance in final interviews. Final pricing was negotiated after selection of the most technically qualified proposal. A procurement agreement for MMS was made on 25 January 2023 with a single manufacturer; this is more than 18 months after implementation of the SCA. About two years later, in December of 2024, the selected manufacturer applied for market authorization of their MMS product. As expected, after regulatory issues had been resolved and the MOH had engaged with the manufacturing community, several other Indonesian manufacturers commenced MMS product development. At the time of Indonesia’s national MMS scale-up launch on 17 October 2024, a total of six manufacturers had begun the application process for market authorization of their product with BPOM.

#### 3.2.2. Tracing Steps to Enable an International Producer to Sell MMS to the MOH and Facilitate Its Importation

The manufacturer selected to trace the steps that needed to be carried out to sell and import MMS (packaged either in bulk for local re-packaging or as a finished product) identified significant, costly challenges to sourcing an imported MMS product, including the need for (1) importation taxes to be paid, (2) a government waiver to import MMS from foreign companies to be signed, (3) a foreign manufacturer to partner with a local manufacturer to serve as an intermediary in order to sell the product to the government (or to allow the foreign manufacturer to create their own locally registered company), and (4) the foreign manufacturer to provide the local partner with the drug master file required for submission of an application to secure market authorization (which is a requirement that foreign manufacturers find universally unacceptable due to the proprietary nature of the information in the drug master file). These requirements make it unlikely for the government to secure optimized pricing for an imported MMS product.

Based on these results, the MOH deemed it feasible/preferable to secure long-term supplies to fill Indonesia’s need for MMS from a local Indonesian manufacturer, assuming two or more qualified manufacturers could obtain market authorization for their MMS product.

### 3.3. Phase 3: MMS-Supply Scale-Up Planning and Implementation

#### 3.3.1. Planning to Scale up a Sustainable Supply of MMS

During phase 3, a multi-year, phased MMS scale-up plan to access MMS supplies was developed and recommended to the MOH. Table 3 illustrates the plan for scaling up MMS in ANC services across Indonesia. In this plan, supplies for a full MMS scale-up over a three-to-four-year period are secured using a combination of donated and MOH-procured MMS. The plan draws on a donor’s commitment to provide MMS supplies and the MOH’s commitment to procure MMS incrementally for much of the remainder of the population of pregnant women served by national health services so that product donation support phases out after year 3. Besides MMS procurement for public sector use, manufacturers are encouraged and expected to ramp up incremental production to sell MMS on both the domestic and export markets. The plan was adopted at the close of 2024 [28].

#### 3.3.2. Plan Implementation

The first step of the scale-up plan included developing and executing a “coaching clinic” hosted by the MOH for manufacturers to (1) clarify their understanding of the Consensus Specification, (2) convey the MOH’s long-term procurement plans, (3) review the regulatory framework for registration and approval of manufacturers’ MMS products, and (4) encourage manufacturers to produce MMS for both domestic and export markets. In year 1 (2025), BPOM started accepting market authorization applications from MMS manufacturers, and the MOH committed to finalizing a tender with pricing for MMS to be purchased by the government in year 2 (2026), subject to manufacturer readiness and MMS manufacturing capacity.

Figure S2 shows how enabling activities occurring in each phase of the investigation were tracked and how each contributed to enabling the supply of MMS, and Table S1 summarizes the key findings regarding enablement tracking by each phase of the investigation. Figure S3 shows the enablement track for supply-related events in relation to the parallel events from a separate study conducted to identify and test a strategy for delivering MMS with high adherence [29]. Figure S3 demonstrates timely convergence of supply and delivery strategies critical to successful MMS introduction and scaling.

## 4. Discussion

### 4.1. Mitigating Risks When Introducing MMS into a National Health System

Introducing MMS into a national health service is an expensive and complex task with risks of delay or failure, but there are ways of mitigating these risks.

#### 4.1.1. Using IS Provides a Framework for Action That Helps Ensure Success

IS provides a comprehensive, evidence-based framework (Figure 1) with which to foster an enabling environment associated with stakeholder awareness and consensus for action that informs strategy development and testing and activities for MMS scale-up. Applying an IS approach provides an overall roadmap that helps foster systematic attention to all activities that need to be performed to ensure timely achievement of success.

#### 4.1.2. Ensuring Stakeholder Coordination/Collaboration Mitigates Risk

Gaining and fostering stakeholder commitment to coordination and collaboration from the outset are difficult tasks to achieve but essential for success. Efficiently securing MMS in a national healthcare system requires coordination and clear/demarcated roles and responsibilities across stakeholders. In Indonesia, this coordination was achieved through (1) government ownership of the initiative and government-led leadership, engagement, and coordination among policymakers, national and international influencers, the MMS-TAG, and technical implementing partners; (2) the use of a lead technical implementing partner to ensure the existence of unified technical, administrative, and financial support (including sub-contracting with a range of partners possessing specialized skills, knowledge, and abilities) for MOH- and MMS-TAG-directed activities; (3) collaboration coordinated by the lead technical implementing partner across all parties.

#### 4.1.3. Maintaining Focus to Prevent Distraction

A stakeholder focus on mission-critical issues relevant to MMS introduction and scaling is requisite for preventing narrow interests from diverting attention and resources. For example, despite unanimous agreement on the efficacy and safety of MMS, numerous concerns were expressed about the feasibility and potential for successful scale-up. Under the guidance of the MMS-TAG with assistance from the lead technical implementing partner, stakeholder concerns converged on the need to identify, simultaneously, (1) an effective delivery strategy focused on assuring high MMS coverage and adherence and (2) an effective strategy with which to secure a sustainable MMS supply for the MOH’s short- and long-term needs. This dual focus was maintained throughout MMS introduction, preventing excursions into activities not central to introducing/scaling MMS.

### 4.2. Policy Lessons and Implications

#### 4.2.1. Securing MMS for Different Program Phases Requires Different Policy and Operational Approaches

The Indonesian Government needed a short-term strategy to secure MMS needed for (1) IR, (2) the period during which legal and regulatory impediments that preclude government purchases of MMS were being overcome, and (3) initial scaling, when MMS delivery, production, and procurement were gearing up. MMS supplies for these needs could be secured via donation of products imported under a research waiver generally available to local universities. Securing MMS to satisfy long-term needs in Indonesia associated with full scaling and maintenance can occur only when MMS is integrated into supply-related policies, the regulatory framework, and institutional support systems (e.g., budget, finance, procurement, and distribution). Other nations can benefit from recognizing that separate policies/operational approaches are needed to secure MMS for short- and long-term needs.

#### 4.2.2. Policy-Related Impediments to MMS Introduction and Scale-Up

In Indonesia, the process of introducing and scaling MMS was mediated in part by (1) sequential efforts (which usually take 4 years to complete) to list MMS on the IEML required to allow MMS use and to list MMS on the IF required for reimbursement for medicines under the national health insurance scheme; (2) the development of an MMS use policy that depends upon IR to identify and test an effective strategy for delivering MMS that can also affect manufacturing requirements (e.g., packaging format and associated tablet counts). Other nations integrating MMS into national supply-related policies should initiate these interconnected actions as early as possible during MMS introduction.

### 4.3. Regulatory Lessons and Implications

#### 4.3.1. Integrating MMS into the Regulatory Framework

Integrating MMS into a national regulatory framework is challenging at several levels but can lead to positive, unintended consequences that strengthen the overall national regulatory function. For new medicines, national drug authorities (e.g., BPOM) need to classify MMS. For nations intending to manufacture MMS for domestic and export markets, Indonesia determined that MMS should be classified as a pharmaceutical product. Subsequently, the focus turned to identifying and making regulatory adjustments to accommodate differences between local pharmacopeial and GMP requirements and internationally recognized standards in the technical specification for MMS. Accomplishing regulatory adjustments required the focused, coordinated effort of experts knowledgeable about the regulations, pharmacopeial and GMP standards, and processes for establishing or amending regulations. While time-consuming, these efforts benefitted the entire regulatory system by triggering a review and update of national pharmacopeial and GMP standards through the lens of internationally recognized standards.

#### 4.3.2. The Value of Adopting Globally Recognized MMS Quality/Manufacturing Standards

When a national drug and food regulatory authority adopts regulatory standards or harmonizes their own with the internationally recognized technical specifications for MMS, value is created for stakeholders (e.g., government, consumers, and manufacturers). Most countries do not possess the minimum market size or technical or manufacturing capacity required for a single national market to produce MMS economically. Nations such as Indonesia (and their local manufacturers) that do meet these requirements will likely encourage MMS production for both national and export markets to more easily achieve manufacturing economies of scale for MMS. Adopting the internationally recognized MMS technical specification (1) promotes “quality equity” for local consumers when MMS meets the standards expected in the international marketplace; (2) promotes convergence among manufacturers to produce a standardized, interchangeable product (e.g., in terms of pharmacopeial and GMP standards); (3) creates a more level economic playing field for manufacturers. Identifying and resolving regulatory issues lead to market success and stakeholder value, whether a nation is an importer or exporter of MMS.

#### 4.3.3. Accommodating the Private Sector in MMS Deployment

When encouraging MMS product development, governments should anticipate shaping the regulatory framework for MMS to accommodate a future commercial role for the private sector to deploy MMS. Manufacturers who consider development of an MMS product will gauge their interests around the channels through which MMS will be permitted to be deployed (e.g., national health services alone, through private-sector channels as over-the-counter sales in retail pharmacies, and social marketing schemes). Indonesia’s MMS regulatory framework anticipated deployment of MMS beyond the national health services to include private sector channels. This was based on recognition that (1) some portion of the public seeks to receive health products/services from the private sector, (2) the marketing power of the private sector could promote MMS use to the benefit of the general public, and (3) granting manufacturers permission to sell the same MMS product through the private sector translates into a powerful incentive for manufacturers to allocate capital investment into MMS product development, provide more favorable pricing of MMS purchased for the national health services, and help deploy MMS to a larger target population, not just those accessing national health services.

### 4.4. Support System Lessons and Implications

#### 4.4.1. MMS Supplies Are Sustainable Only if Integrated into All Support Systems

Whether a nation procures its own MMS, is dependent on donated MMS products, or uses some combination of both, the most overlooked part of securing a sustainable supply of MMS is integrating it into all institutional support systems—i.e., budget, finance, procurement, and distribution. Of these support systems, integrating MMS into budget and finance systems is the most challenging and time-consuming to achieve.

The MOF and the MOH in Indonesia assessed affordability and the case for adoption of MMS differently. The MOF was concerned about whether MMS requires new financial resources, whereas the MOH examined the cost–benefit ratio of MMS to justify its introduction into the national health system. These positions are likely replicated in other countries. Creating a budget impact analysis was one of the most important and effective ways to help successfully persuade the MOF to allocate financial resources required to enable ongoing and sustained access to MMS supplies. Although donor financing helped the MOH to introduce and scale MMS use in ANC services in Indonesia, it could not substitute for the national political will to budget for MMS use as a sustainable, long-term investment in any national ANC service.

Financing MMS is a key long-term challenge for national governments intending to scale up and maintain MMS. Indonesia has multiple avenues for financing long-term MMS product costs. Sustained commitment of national revenues is critical to MMS scale-up and the maintenance of MMS deployment. Although the Child Nutrition Fund/UNICEF [30] and World Bank lending for nutrition mechanism [31] and product donations help implement MMS, it is important to recognize that in the future, such support may only be available to countries in greatest need. Governments are challenged to establish their own priorities and financing for long-term MMS procurement, as achieved by Indonesia.

#### 4.4.2. Integration of MMS into Procurement and Distribution Systems Is the Last Step in Securing a Long-Term Supply of MMS on a Sustained Basis

Procurement and distribution systems generally cannot be activated until MMS is fully integrated into supply-related policies, the regulatory framework, and budget/finance support systems. Each country has its own unique procurement rules that must be met before regularized procurement and distribution can begin. It is important for decision-makers to recognize the importance of integrating MMS into these systems to ensure and secure a long-term supply of MMS on a sustained basis.

### 4.5. MMS Manufacturer/Supplier Lessons and Implications

#### 4.5.1. Achieving a Commercially Viable Product Takes Years and Requires Technical Inputs

Typically, skilled Indonesian manufacturers (who had never produced MMS) took three to six months to formulate an MMS product and another twelve to eighteen months to demonstrate acceptable product stability consistent with local and international regulatory requirements. Achieving a viable product also required significant technical assistance for producers, especially with respect to achieving pharmacopeial and GMP standards because of the complexity of MMS manufacturing and testing.

Once manufacturers had a viable product, it took several months to secure market authorization, and it will take anywhere from several months to years to scale manufacturing capacity to meet national and export needs. Experience suggests MMS buyers should recognize that new manufacturers will not have optimized manufacturing capacity (and thus higher initial pricing) and will seek to understand MMS demand before scaling production. Thus, manufacturers will seek terms and conditions for purchase agreements to allow them to grow their capacity over time. Manufacturers tend to plan initial capacity at the lower end of manufacturing cost-efficiency (e.g., MMS for about 1 million pregnancies annually) and scale capacity thereafter [32,33].

#### 4.5.2. Local Manufacturing or Importation of MMS—That Is the Question

This is the most asked question by policy- and decision-makers. Indonesia had good reasons to explore locally manufacturing MMS instead of importing it. Nevertheless, national officials assessed both so that the nation had options. Apart from the key indicators used to anticipate manufacturer potential as a supplier (shown in Table 1), the practical considerations driving decision-making in Indonesia were whether (1) the nation had a sufficient number of pregnancies each year to support cost-efficient manufacturing; (2) there were at least two qualified manufacturers able and willing to produce and sell a high-quality, low-cost MMS product to the national government to avoid sole-source contracting; (3) the national government was willing to exempt MMS ingredients and/or the finished product from all or most import taxes; (4) manufacturers were interested in selling it on the export market and had the physical capacity to expand production to achieve optimal manufacturing cost-efficiency (typically reached when producing MMS for at least 5 million pregnancies per year) [33]. Experience in Indonesia across these considerations suggests that few nations will be able to grow their local MMS manufacturing capacity on a practical and cost-efficient basis.

#### 4.5.3. Donor Assistance Can Be Critical in Effectuating Local Manufacturing

The key factors accelerating progress in securing a sustainable supply of MMS from within Indonesia were offers from a private foundation to (1) issue a purchase agreement to a local manufacturer when the government was legally unable to do so and (2) provide ongoing technical assistance to the manufacturer, ensuring their product conformed with the Consensus Specification. These circumstances were unplanned at the outset of this investigation and unlikely to be available to other nations. Nevertheless, the foundation’s offer catalyzed manufacturing in Indonesia and eventually encouraged other Indonesian pharmaceutical manufacturers to begin product development.

### 4.6. Study Limitations and Transferability of IS Methods to Other Settings

Other countries applying IS to secure a sustainable supply of MMS will face limitations regarding transferability, especially those with small populations with under 1 million pregnancies/year and smaller economies. The systematic approach of IS and the tools developed or otherwise adapted for this investigation provide a robust evidence-based process for generating information that can promote awareness-raising, consensus-building, and decision-making needed in any setting to achieve (1) integration of MMS into supply policies, a regulatory framework, and support systems required to sustain MMS in ANC services and (2) scaling of MMS services. The IS process and tools can also yield important insights into the technical and economic viability of local MMS manufacturing in any setting.

However, it may be unrealistic to conduct IS phase 2 (i.e., testing alternative strategies for sourcing MMS via importation or local manufacturing) outside a select few countries. This limitation is attributable to (1) factors discovered during landscaping that result in a default conclusion that local manufacturing is not technically or economically viable (e.g., countries with few annual pregnancies, a limited pharmaceutical manufacturing infrastructure and skilled workforce, and a manufacturer’s inability to sell, competitively, to an export market that contributes to manufacturing efficiency), (2) legal barriers that prevent a government from conducting test procurements (i.e., MMS has not yet been integrated into supply policies, the regulatory framework, or the support system needed to trigger government procurement action), (3) government resistance to enter into advance purchase agreements with advance payment to help finance product development for an entirely new product requiring three to four years to yield a qualified product for purchase, and (4) disinclination of donors to finance development of MMS manufacturing unless it contributes credibly and meaningfully to the global supply of MMS produced to internationally recognized product standards.

## 5. Conclusions

Introducing a new intervention such as MMS into national health services is a complex task that requires support from the private, non-profit, academic, and government sectors. Securing a sustainable supply of MMS requires the creation of new national supply-related policies; a regulatory framework for guiding MMS development, production, and market authorization; and integration of MMS into national budget, finance, procurement, and distribution systems. Applying IS provides a systematic, evidence-based framework and methods that include (1) information gathering, sharing, and consensus-building required to create an enabling environment in order to identify and understand sustainable sourcing options; (2) evaluating MMS supply strategy options; (3) planning and executing the scale-up of an MMS *supply* strategy informed by IR and synchronized with scaling up the deployment of MMS using an effective MMS *delivery* strategy. The experience gained from these efforts yielded important lessons with implications for other countries choosing to explore MMS and introduce it into their national health systems.

## Figures and Tables

**Table 1:** Key indicators used to determine a manufacturer’s potential as a supplier.

Manufacturers’ evidence and/or documentation of their Experience blending at least 10 active ingredients; Ability to produce MMS according to internationally recognized quality standards and WHO pharmaceutical GMP guidelines consistent with the Consensus Specification; Ability to produce an MMS product with a minimum 30-month shelf life under ICH climatic zone IVb conditions; Ability to produce a halal-certified MMS product; Ability to manufacture at least 180 million MMS tablets packaged in 180-count bottles (sufficient for at least one million pregnant women) annually, with demonstrated potential for scalable production; Ability to self-finance product development and equipment upgrade costs; Experience selling drug products in the international marketplace; Willingness to sell a product to the Indonesian MOH.

**Table 2:** The results obtained from landscaping the supply policy, supply regulatory, and manufacturer/supplier contexts [20,21,22,23].

Themes	Findings	MMS-TAG Recommendations Made to the MOH	MOH Enabling Actions/Outputs
**Section 2.1: Supply Policy Context**			
Lack of policies permitting MMS procurement/access	Medicines dispensed by the MOH must be locally manufactured No policies provide a legal mechanism for MMS procurement; exceptions are granted to research universities	Permit universities to import donated MMS for IR Hire pharmaceutical experts to examine sourcing options (local production vs. importation) for securing long-term MMS supply	Allowed use of donated/imported MMS for short-term IR needs Activated technical expertise from PHARCI to explore options to secure MMS for long-term needs Put plans in place to adopt policies permitting long-term MMS procurement
2. Absence of MMS listing on IEML and IF	Medicines dispensed by the national health service (NHS) must be listed on the IEML and the IF No policies to permit deployment of MMS	Leverage existing processes to list MMS on the IEML and IF	PHARCI mapped processes enabling MMS registration on IEML and IF that can be put into place once the manufacturer’s application for market authorization is approved by BPOM Use of donated MMS approved under “special access scheme”
3. Absence of a formal designated classification of MMS	Medicines (including supplements) used in the NHS must be classified Supplements with any active ingredient >1 RDA must be classified as “health supplement”	MMS must be classified as a health supplement but manufactured as a pharmaceutical product under USP pharmacopeial standards and WHO GMP guidelines to ensure its acceptance in export markets	The MOH affirms that UNIMMAP MMS must be treated as a health supplement and, specifically, manufactured as a pharmaceutical product under USP pharmacopeial standards and WHO pharmaceutical guidelines
4. Lack of policies affecting service providers’ use of MMS	NHS medicine use is guided by “use” protocols for service providers, but there are no MMS use protocols Related MMS policy questions should be addressed (e.g., how can MMS adherence be improved; are there allowable uses of MMS among non-pregnant women; how should MMS be used in contexts of anemia for treatment versus prevention; and what MMS packaging option should be adopted (e.g., 30-, 90-, and 180-count bottles or blister packs))	Take action to shape and adopt service provider use policies Shape and adopt service provider “use” protocols Collaborate with academic partners to answer related policy questions via IR Communicate policies for service provider “use” protocol based on IR findings and global guidance	MOH directed activation of processes to create MMS “use” protocols for service providers and IR for related policy questions National MMS use protocols for service providers were adopted in 2024 [20]
**Section 2.2: Supply Regulatory Context**			
Internationally accepted UNIMMAP MMS formulation is at variance with Indonesian regulations and Indonesian pharmacopeial standards for selenium	UNIMMAP MMS contains 65 mcg of selenium, while Indonesian supplement regulations only permit a maximum of 60 mcg of selenium for use in health supplements for pregnant women, creating a question about whether MMS can be registered for commercial sale and use in Indonesia	Convene a series of national scientific meetings to be attended by experts from the MOH, BPOM, and academia (local and international) to review the maximum level of nutrients permitted for use in health supplements in Indonesia and internationally	Expert meetings were held to review, discuss, and generate opinions on increasing the allowable level of selenium from 60 mcg to 65 mcg BPOM conducted an extensive review of regulations/evidence regarding selenium use in health supplements for pregnant and breastfeeding women A UNIMMAP MMS formulation with 65 mcg of selenium was adopted and codified by the Minister of Health in 2024 for public and private sector use [20], and the Director General of Primary and Community Health subsequently issued a decree mandating the integration of the UNIMMAP formulation into the Technical Specifications of Micronutrient Supplements for Pregnant Women under the Special Program [21] BPOM adjusted regulations to align its maximum daily intake for selenium for pregnant women with the internationally accepted UNIMMAP formulation containing 65 mcg [22]
2. International MMS quality and GMP standards are at variance with Indonesia’s regulatory standards for health supplements	BPOM tests only index vitamin levels, while the internationally recognized standard requires all nutrients be tested BPOM lacks a requirement to conduct dissolution testing, except for controlled release products; the internationally recognized standard requires dissolution testing for four nutrients: vitamin A, folic acid, vitamin B2, and iron For medicines not in the Indonesian Pharmacopeia—e.g., oil and water-soluble vitamins with minerals tablets—pharmacopeial standards applied must be any internationally recognized pharmacopeia according to BPOM regulation No. 24 of 2024 [23]. Thus, BPOM’s regulation for the safety and quality of health supplements can rely on the internationally recognized USP Pharmacopeia.	Accept independent expert evaluation findings made by PHARCI Accept internationally recognized pharmacopeial and GMP standards contained in the Consensus Specification with modest non-material adaptations to accommodate certain requirements in the Indonesia Pharmacopeia	The MOH adopted the internationally accepted Consensus Specification with some adaptation for the Indonesian context to clarify BPOM’s regulatory guidance for MMS; BPOM officially endorsed/adopted these recommendations [21] BPOM’s acceptance of the internationally recognized Consensus Specification clarified the legal basis and provided technical guidance for local pharmaceutical companies with respect to developing, producing, and registering MMS for use in MOH programs and services
**Section 2.3: Manufacturer/Supplier Context**			
Enablers of local manufacturing	Increasing global MMS implementation stimulates manufacturers’ interest in producing MMS for and selling it to domestic and export markets Indonesia’s population size can support multiple local manufacturers in producing MMS profitably and at a low cost for the MOH There are attractive business opportunities for manufacturers to (1) engage in retail sales of an identical MMS product that could encourage concessionary pricing of MMS with respect to the MOH and (2) open new private-sector markets for MMS to serve women other than pregnant women (e.g., breastfeeding and pre-conceptual women) The Ministry of Planning is seeking Indonesian manufacturers to produce MMS for export	Collaborate with technical partners to test/pilot local manufacturing while continuing to explore importation of MMS as a backup plan Continue to identify/implement enabling actions leading to resolution of regulatory issues impeding manufacturers’ interest in producing MMS Acknowledge that local manufacturing is feasible, but scale-up efforts must recognize that (1) a locally manufactured MMS supply will take a minimum of three years to gain market authorization, (2) significant technical manufacturing and regulatory issues will need to be overcome, (3) a backup plan is needed in case local manufacturing does not succeed, and (4) local manufacturing capacity will take time to ramp up to satisfy the demand for the entire eligible population, thus impacting the duration for which imported products will be required for scaling	The MOH put plans in place to evaluate local manufacturing capabilities while exploring importation of MMS as a backup plan The MOH leveraged ongoing importation of donated MMS to fulfill short-term needs for MMS for an extended period while local manufacturing was scaling up The MOH approved activities to explore both importation and local manufacturing to fulfill long-term scaling and maintenance needs See results in the supply policy context and regulatory context sections of this table for enabling actions and outputs pertaining to resolving policy and regulatory issues impeding local manufacturers’ interest in producing MMS
2. Barriers to local manufacturing	Manufacturers are hesitant to undertake product development without an advanced purchase agreement Manufacturers unable or unwilling to share information about manufacturing capacity There is resistance to investing in multi-year product development without a regulatory framework for registration, approval, and use of MMS in Indonesia There is concern about operating competitively in domestic and international marketplaces without an internationally harmonized standard for manufacturing MMS There is uncertainty about how import taxes on ingredients might affect pricing		

**Table 3:** Scaling-up plan.

Commitment	Year 1	Year 2	Year 3	Year 4	Year 5
MOH	Tender prepared	1/3 of pregnancies		2/3 of pregnancies	88% of pregnancies
Donor support	1/3 pregnancies			None (donor support is phased out as local manufacturing begins)	
Private sector	None			Introduction into retail pharmacy chains	12% of pregnancies

## Data Availability

The original contributions presented in this study are included in the article/Supplementary Material. Further inquiries can be directed to the corresponding author. Data generated in connection with the manufacturers’ assessments were obtained confidentially to protect any proprietary or sensitive business information provided. Data for the enablement figures and non-proprietary information pertaining to policy, regulations, and support systems were generated and presented as described. Requests for data will be considered on an individual basis; proprietary information about the manufacturers will not be shared.

## Supplementary Materials

The following supporting information can be downloaded at: https://www.mdpi.com/article/10.3390/nu18132162/s1, Figure S1: The Indonesian Multiple Micronutrient Supplementation Technical Advisory Group (MMS-TAG); Figure S2: Cumulative enablement track for all key IS Phase 1-3 events; Figure S3: Cumulative enablement track for all Supply and Delivery events; Table S1: Enablement tracking key findings by phase of investigation.

## Abbreviations

The following abbreviations are used in this manuscript:

ANC	Antenatal care
BPOM	Badan Pengawas Obat dan Makanan (The Indonesian Food and Drug Authority)
BSPH	Johns Hopkins University Bloomberg School of Public Health
FGD	Focus group discussion
GMPs	Good manufacturing practices
IGI	Inisiatif Gizi Indonesia (Indonesian Nutrition Initiative)
IFAS	Iron and folic acid supplementation
IEML	Indonesian Essential Medicines List
IF	Indonesian Formulary
IR	Implementation research
IS	Implementation science
LMIC	Low- and middle-income countries
Mcg	Micrograms
MOF	Ministry of Finance of the Republic of Indonesia
MOH	Ministry of Health of the Republic of Indonesia
MMS	Multiple-micronutrient supplementation or supplements
MMS-TAG	Indonesian Multiple Micronutrient Supplementation—Technical Advisory Group
PHARCI	Center of Excellence for Pharmaceutical Care Innovation, Universitas Padjajaran
RDA	Recommended daily allowance
RFP	Request for proposal
SCA	Supply Context Assessment
UNICEF	United National Children’s Emergency Fund
UNIMMAP	United Nations International Multiple Micronutrient Antenatal Preparation
WHO	World Health Organization
