# Supplementary material for: Applying Implementation Science to Secure a Sustainable Supply of UNIMMAP MMS for National Antenatal Care Services in Indonesia
Source: Nutrients. 2026 Jul 3;18(13):2162. doi: 10.3390/nu18132162 (PMC13363487; doi:10.3390/nu18132162)
Supplement: Supplementary file 1 [file nutrients-18-02162-s001.zip › nutrients-4190896-supplementary.pdf]

Nutrients Manuscript ID: nutrients-4190896. Corresponding Author: Clayton Ajello
Supplementary Materials: Tables & Figures in the order of their reference in the paper: Figure S1, Figure S2, Table S1, and Figure S3

Figure S1. The Indonesian Multiple Micronutrient Technical Advisory Group (MMS TAG)

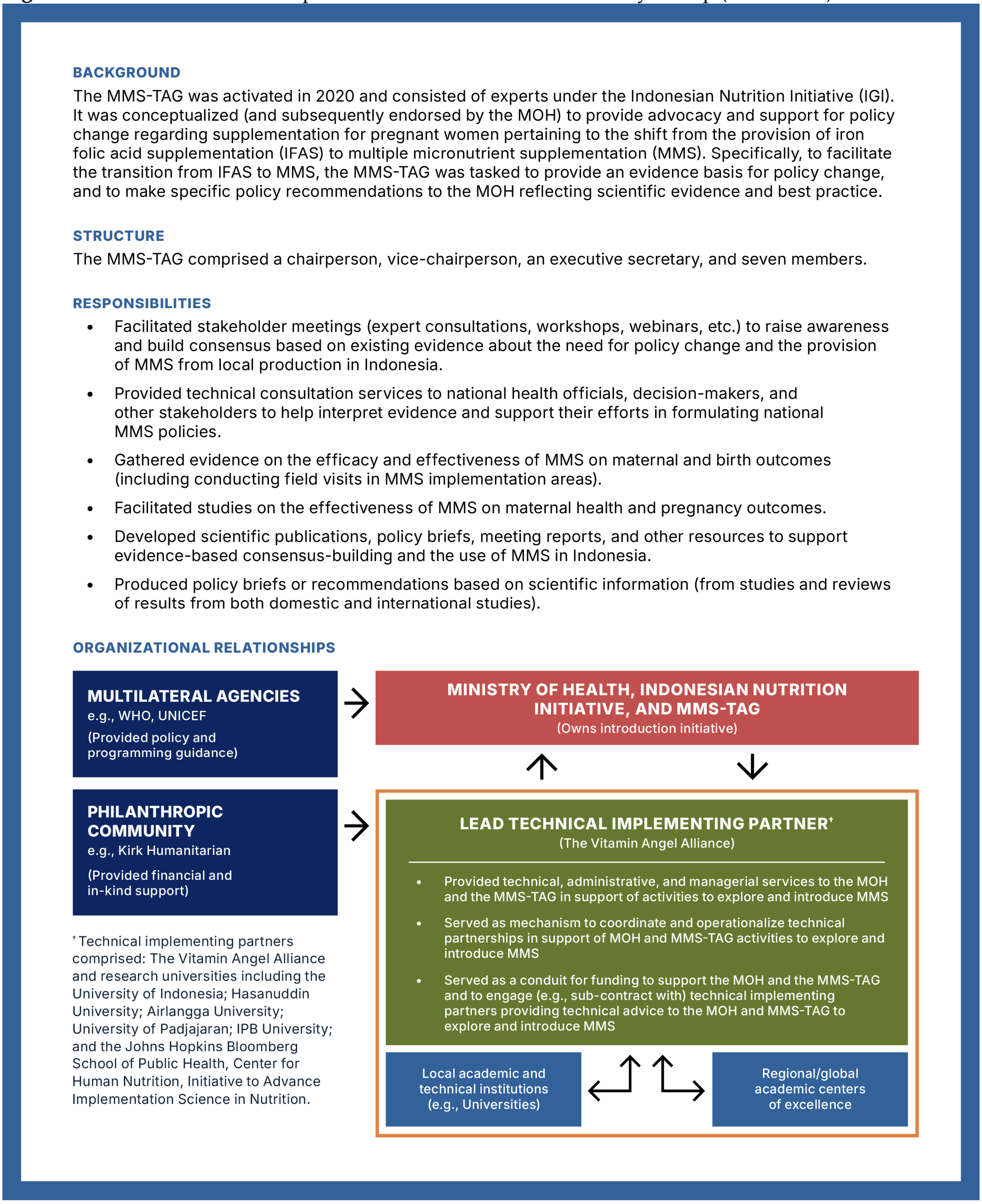

Figure S2. Cumulative enablement track for all key IS Phase 1-3 events

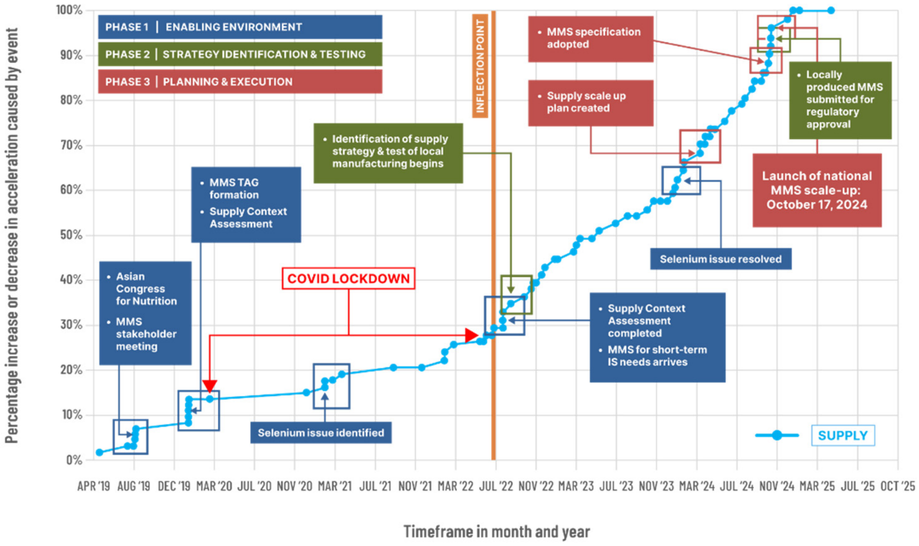

The enabling activities performed throughout this investigation, recorded and ranked for their contribution to enabling a sustainable supply of MMS and plotted according to when they occurred, are illustrated in Figure S2.

Stakeholder rankings were generated through a structured scoring and ranking exercise systematically generated by engaging only stakeholders who were directly involved in or had sufficient knowledge of an event and invited to provide an assessment of that event. Events, including date, participants, written description, and images were documents prospectively, and ratings were assigned retrospectively by participants during a workshop held on May 5, 2025. Participants were asked to provide an assessment of each of the 95 events in the form of 1) an acceleration score of +1 to +10 that represented the event's influence on increasing the transition speed to MMS (e.g., catalytic, pushed forward, resulted in consensus or next steps), 2) a deceleration score of -10 to -1 that represented the event's influence on decreasing the transition speed to MMS (e.g., delayed progress, required additional steps, caused confusion), 3) a neutral score of 0 that represented that an event neither increased nor decreased the transition speed to MMS, or 4) don't know.

Individual scores were then aggregated across all respondents to generate a ranking at the event level. Subsequently, raw scores were transformed into proportional values on a scale of 0% to 100%, enabling a comparison of each event's relative contribution to the MMS scaling process. A higher percentage indicates that an event is perceived to have a stronger influence in accelerating the transition process, while a lower percentage indicates that the event is perceived to have a weaker influence or even to slow down the transition process.

Each of the 95 dots in Figure S2 represents a separate event or activity performed between 2019 and 2024 to support the introduction of MMS and reflects the time of the event's occurrence on the x-axis and the relative strength of its contribution to progress towards securing a sustainable supply of MMS on the y-axis. A summary of results depicted in Figure S2 are described in Table S1.

Table S1. Enablement tracking key findings by phase of investigation

**Phase of Investigation**	**Key Findings**
Phase 1	• Covid lockdown caused uncertain effects on progress given context landscaping and enabling activities continued largely uninterrupted<br>• Fostering an enabling environment required constant attention and action across the 5-year period<br>• The slope of the enablement track during most of phase 1 and up to the completion of the SCA (marked by the vertical inflection point line shown in Figure 2) is relatively flat, after which the enabling environment progressed at more rapid pace<br>• 30 months elapsed between the time the regulatory problem with the level of selenium in MMS was identified and resolved
Phase 2	• It took 32 months from the time a supply strategy and a local manufacturer were identified to when an experienced, local manufacturer was able to complete development of an MMS product and apply for regulatory approval (i.e., market authorization)<br>• Local submission of MMS for market authorization and approval of the application demonstrated feasibility of local manufacturing an MMS product
Phase 3	• Supply scale-up planning and initial execution with related enablement activities were achieved in a relatively short six-month period ending with the official launch of the national MMS scale-up plan on October 17, 2024
Overall	• Supply events conducted during all three IS phases did not occur sequentially but overlapped in time<br>• While fostering an enabling environment in phase 1 took the longest time to complete, it created significant momentum that led to accelerated progress in phases 2 and 3 of this investigation

Figure S3. Cumulative enablement track for all Supply and Delivery events

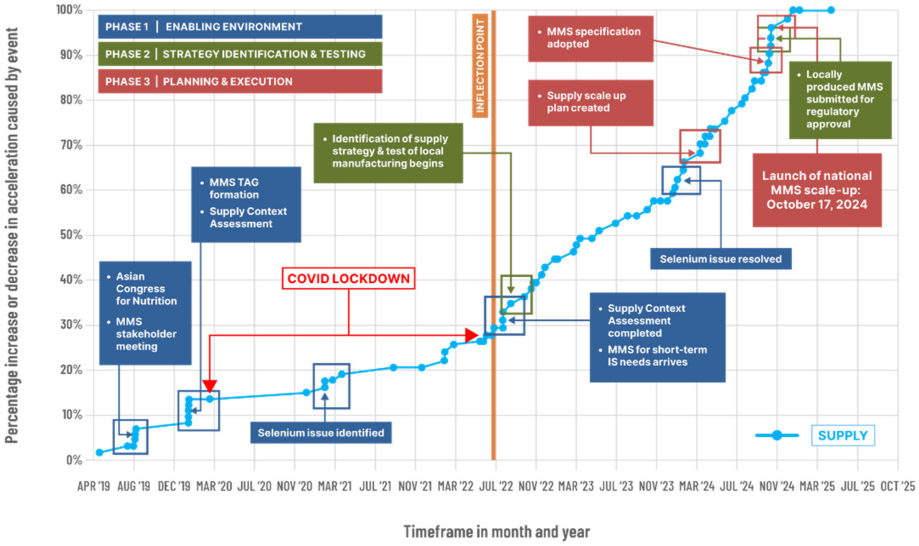

Figure S3 shows the enablement track for all supply events in relation to events from a separate study conducted to identify and test a strategy to achieve delivery of MMS with high adherence. Figure 3 shows that both supply and delivery enablement tracks converge very rapidly in the 6 months ending with the official launch of MMS scaling. This convergence would not have occurred without the MMS-TAG and full support of the MOH focused simultaneously on developing both effective supply and delivery strategies. Realizing this convergence is an essential desired outcome for every country introducing MMS into their national health services, especially because securing a new medicinal product, which is often assumed to be readily available, is the rate limiting step to scaling-up its delivery.
